# Supplementary material for: The interactome of intact mitochondria by cross-linking mass spectrometry provides evidence for coexisting respiratory supercomplexes
Source: Mol Cell Proteomics. 2017 Dec 8;17(2):216–32. doi: 10.1074/mcp.RA117.000470 (PMC5795388; doi:10.1074/mcp.RA117.000470)
Supplement: Supplemental Data [file supp_RA117.000470_133922_0_supp_23978_fzffwf.zip › spectra_annotation/mito_spectra_annotation/493-1-6-1-7-1.pdf]

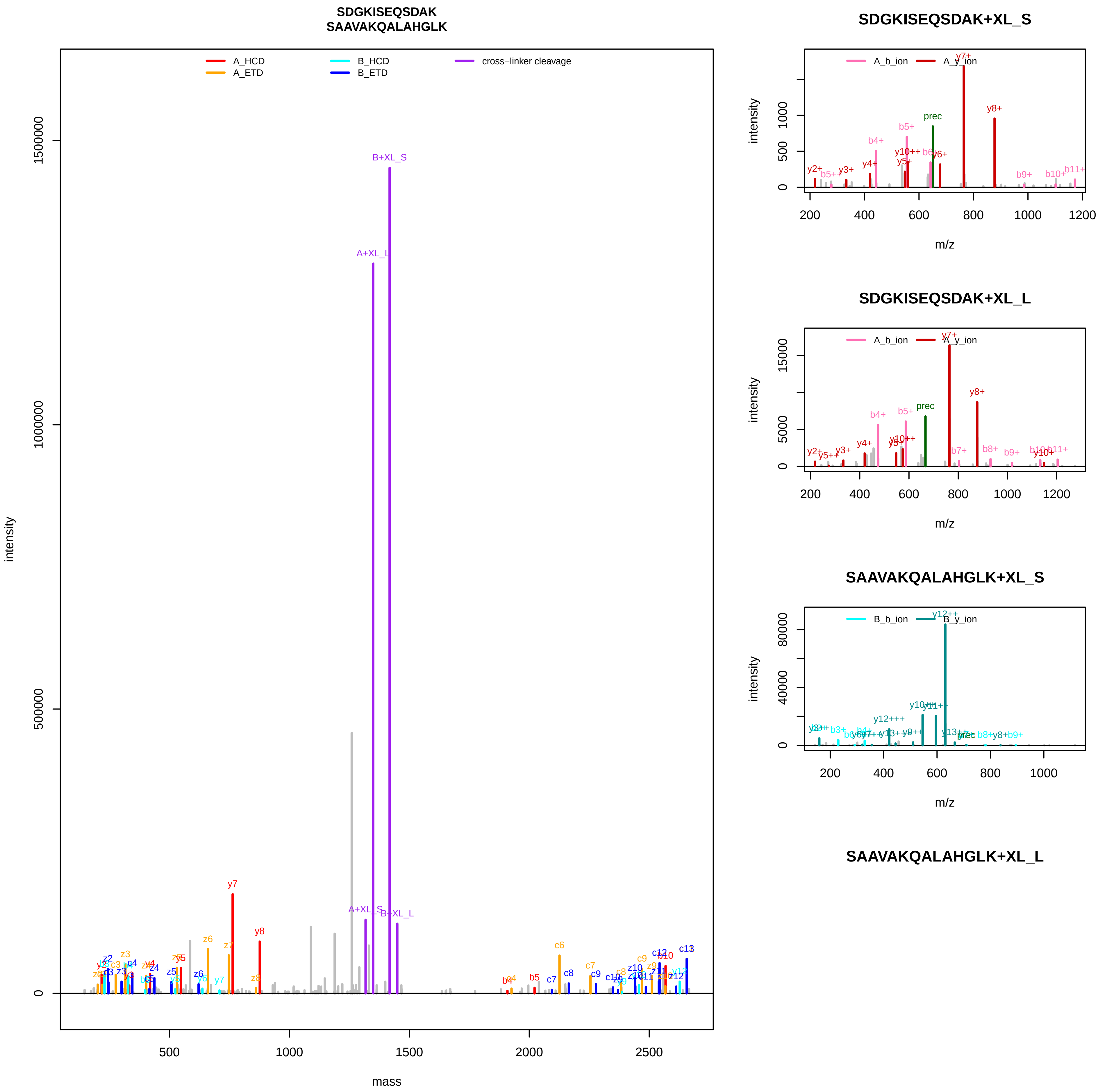
SDGKISEQSDAK
SAAVAKQALAHGLK
A_HCD
A_ETD
B_HCD
B_ETD
cross−linker cleavage
B+XL_S
A+XL_L
A+XL_S
B+XL_L
y7
y8
b4
b5
c6
c7
c8
c9
c13
intensity
mass
SDGKISEQSDAK+XL_S
A_b_ion
A_y_ion
prec
y7+
y8+
b5+
b4+
y2+
y3+
y4+
y5+
y6+
b9+
b10+
b11+
m/z
SDGKISEQSDAK+XL_L
b7+
b8+
y10+
SAAVAKQALAHGLK+XL_S
B_b_ion
B_y_ion
y12++
y11++
y12+++
b3+
b4+
y8+
b9+
SAAVAKQALAHGLK+XL_L
